# mRNA-Based Vaccine for COVID-19: They Are New but Not Unknown!

**DOI:** 10.3390/vaccines11030507

**Published:** 2023-02-22

**Authors:** Vivek P. Chavda, Gargi Jogi, Srusti Dave, Bhoomika M. Patel, Lakshmi Vineela Nalla, Krishna Koradia

**Affiliations:** 1 Department of Pharmaceutics and Pharmaceutical Technology, L M College of Pharmacy, Ahmedabad 380009, India; 2 Department of Pharmacology, Institute of Pharmacy, Nirma University, Ahmedabad 382481, India; 3 School of Medico-legal Studies, National Forensic Sciences University, Gandhinagar 382007, India; 4 Department of Pharmacy, Koneru Lakshmaiah Education Foundation, Vaddeswaram 522302, India; 5 Department of Pharmaceutics, Saurashtra University, Rajkot 360005, India

**Keywords:** COVID-19 vaccine, mRNA vaccines, vaccine development, vector-based vaccines

## Abstract

mRNA vaccines take advantage of the mechanism that our cells use to produce proteins. Our cells produce proteins based on the knowledge contained in our DNA; each gene encodes a unique protein. The genetic information is essential, but cells cannot use it until mRNA molecules convert it into instructions for producing specific proteins. mRNA vaccinations provide ready-to-use mRNA instructions for constructing a specific protein. BNT162b2 (Pfizer-BioNTech) and mRNA-1273 (Moderna) both are newly approved mRNA-based COVID-19 vaccines that have shown excellent protection and efficacy. In total, there are five more mRNA-based vaccine candidates for COVID-19 under different phases of clinical development. This review is specifically focused on mRNA-based vaccines for COVID-19 covering its development, mechanism, and clinical aspects.

## 1. Introduction

Coronavirus disease-19 (COVID-19) pandemic caused by severe acute respiratory syndrome coronavirus-2 (SARS-CoV-2) has spread worldwide. As of now, globally, there have been 662 million confirmed cases of COVID-19, including 6.6 million deaths reported to the World Health Organization (WHO) [1]. The virus affects more than 200 countries worldwide, with the bulk of cases reported in Brazil, Russia, and the United States [2]. The first report of an outbreak of COVID-19 was from Wuhan city, Hubei Province of China on 30 December 2019. Clusters of cases of pneumonia were recorded in Wuhan. Later on 7 January 2020, a novel coronavirus (2019-nCoV) was found to be the causative agent. The WHO eventually named the disease COVID-19 [3,4]. The estimated case fatality rate ranges from 0.5–1.5%. SARS-CoV-2 belongs to the family *Coronaviridae* and the order *Nidovirales*. SARS-CoV-2 belongs to Betacoronavirus having a close genomic relation with two viruses, SARS-CoV and Middle Eastern respiratory syndrome coronavirus (MERS-CoV). As compared to SARS and MERS, SARS-CoV-2 is highly contagious. COVID-19 is a serious illness of global significance. The illness cannot be treated with a specific antiviral medication, the key to preventing the spread of the disease is to break the chain of transmission [5]. To decrease the prevalence and mortality rates, mass immunization against the pandemic is used [6,7]. Since the SARS-CoV-2 genome sequence was released on 11 January 2020, more than 150 authority immunization projects were studied. The main goal of vaccination is to induce an immune response that provides long-term protection against severity of the disease [8]. As evaluated by Paddy Ssentongo and colleagues [9], vaccine efficacy (VE) against all SARS-CoV-2 infections fell from 83% in the first month after completion of the first immunisation series to 22% after 5 months or more. Similarly, VE against symptomatic COVID-19 decreased from 94% in the first month to 64% during the fourth month following immunisation. Overall, VE against severe COVID-19 was strong at all ages, with a level of 90% (95% CI, 87–92%) five months or more after being completely vaccinated.

Coronavirus has spiked (S) protein on its surface; this S protein will interact with the receptor-binding domain of the angiotensin-converting enzyme (ACE) receptor [10,11]. Most of the vaccine candidates are targeting this S protein of the virus [12]. The Pfizer-BioNTech COVID-19 Vaccine-BNT162b2 is the first immunization approved by the Food and Drug Administration (FDA). It is based on messenger RNA (mRNA) made by Pfizer which received emergency use authorization for 12–15 years of age or older for the prevention of the COVID-19 disease. US food and drug administration (USFDA) approved the Pfizer vaccine on 23 August 2021 [13]. Various studies have reported that immunization is having an efficiency of 89.1% to prevent the SARS-CoV-2 infection; additionally, vaccines have also decreased COVID-19-related hospitalization, deaths, and prevention of ICU admission. Controllable spread of SARS-CoV-2 can be accomplished earlier when a large proportion of the population is immunized (e.g., 70–80%). Immunization significantly reduces the incidence of COVID-19. The vaccine provides a robust and effective immune response to eradicate the SARS-CoV-2 infection from the human body. People who were infected had a potent T-cell response to the virus, which may aid in their ability to recover from the infection [14,15]. Neutralisation remained strongly correlated with protection from symptomatic infection with SARS-CoV-2 variants of concern [16]. Thus, vaccination is the strategy to control the pandemic. 

**Figure 1:**
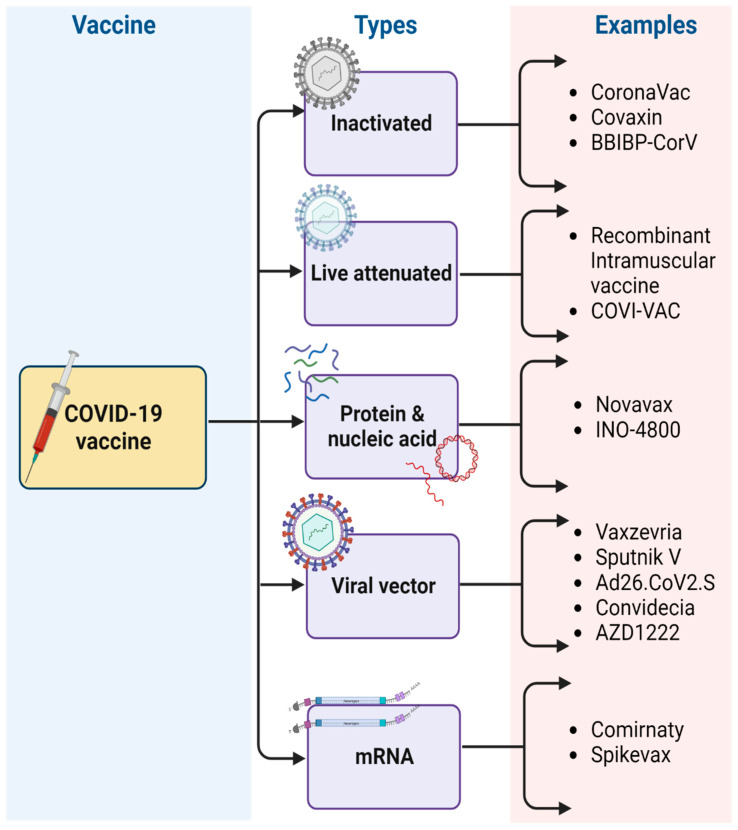
Different types of vaccine for COVID-19 infection. (Created with BioRender.com accessed on 31 December 2022).

COVID-19 vaccines are being developed on a variety of platforms. A live attenuated virus, inactivated vaccines, non-replicating viral vectors, RNA, and DNA are examples of these. Different types of vaccines with examples are listed in the Figure 1. A strategy for vaccine development is of using the killed SARS-CoV-2 virus, it is not able to replicate but it can remain intact in the body. The immune system will trigger its response against the virus by making antibodies against it. COVAXIN^®^ (BBV152) manufactured by Bharat Biotech, Hyderabad, India, and CoronaVac also known as sinovac^®^ developed by Sinovac Biotech, Beijing, China has used inactivated virus approach to develop the vaccine [17]. The nonreplicating viral vector is also a technique adopted by companies for vaccine production. The adenovirus (Ads) is a widely used viral vector that has a double-stranded genome acting as a causative agent for a common cold. CanSino Biologics Tianjin, China has developed Ad5-nCoV (Convidecia^®^) which encodes the full length of S-protein of the SARS-CoV-2 virus. The viral vector does not possess replicating properties and hence does not cause actual disease. Once entered into the body the viral DNA is presented on antigen-presenting cells and an immune response is generated against it. Oxford/Astrazeneca has used adenovirus from the chimpanzee (ChAdOx1) which possesses the potential to minimize interaction with prevalent antibodies against adenovirus. The vaccine is named AZD122 (Vaxzevria™). The Gamaleya Research Institute, Moscow, Russia developed the Sputnik V™ vaccine is also a viral vector having recombinant human Ad26 serotype. The Janssen vaccine was manufactured by Johnson and Johnson, New Jersey, USA using the same approach [18]. 

mRNA is also considered a new approach to formulating a vaccine. mRNA vaccines are new to the public, but scientists have been researching them for a long time. These mRNA vaccines are synthesized from the DNA template which encodes the spike protein and is packed into the lipoprotein-based carrier to speed up the entry of the mRNA inside the body and prevent degradation [19]. When the vaccine is given through the intramuscular route, it is injected into deeper tissues and the mRNA molecules will enter inside the cell, facilitating the translation process [20]. Upon entering the body, mRNA will be recognized as an antigen and a humoral immune response will be activated which will stimulate B cells to develop into memory B cells. Thereby on secondary exposure to the antigen; memory B cells will neutralize and block the antigens [21]. FDA has approved mRNA-based vaccines, one of which is Pfizer-BioNTech’s, New York, NY, USA BNT162b2 (Comirnaty^®^) and another one is Moderna™, Cambridge, MA, USA mRNA-1273 also known as Spikevax. The FDA approved mRNA-1273 for emergency use authorization (EUA) for use in people aged 18 years and over. The current review will provide detailed information about vaccinology and facts about COVID-19, mRNA-based vaccine development, how mRNA vaccines work, the formulation and ingredients used, safety and efficacy, and the regulatory challenges for vaccines. 

## 2. Vaccinology and Facts about COVID-19

**Figure 2:**
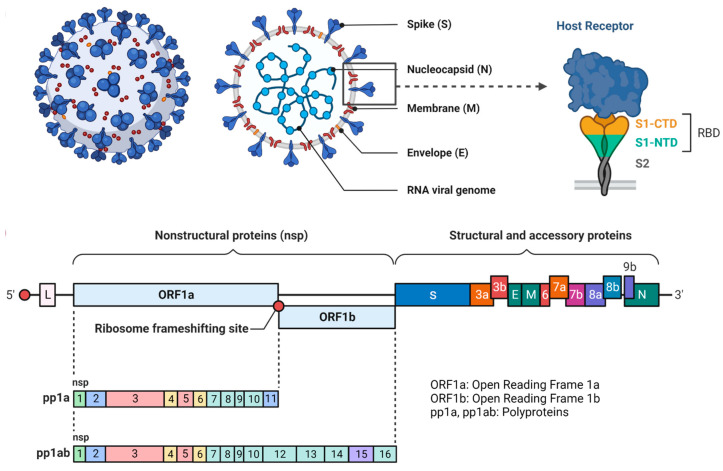
Genomic structure of coronavirus. (Created with BioRender.com accessed on 31 December 2022).

In December 2019, a group of individuals suffering from lung disorders for an unidentified reason was reported in China. The Chinese Centre for Disease Control and Prevention (CDC) then investigated the uncertainty in pneumonia-related cases and their origin by collecting specimens from the patient’s body which were detected using real-time polymerase chain reaction [22]. Later, the cause of the lung disorder was known and it was found to be caused by a coronavirus, which was further named SARS-CoV-2 (severe acute respiratory syndrome) or COVID-19. Since then, it has turned into a global pandemic. It belongs to a group of β-coronaviruses. Structurally, it is an enveloped, non-segmented, belonging to the subgenus of *Sarbecovirus,* and *Orthocoronavirinae* subfamily. The genomic material present in its structure is the single-stranded positive-sense RNA sequence [23]. They are found primarily in avian and mammal hosts. The genomic structure of coronavirus has been depicted in Figure 2, which represents that the viral structure possesses spike proteins, having subunits, S1 and S2, on the outer membrane. The RNA viral genome is encapsulated inside the membrane along with the nucleocapsid. Thus, we can say that there are four structural proteins, i.e., the spike protein (S), nucleocapsid protein (N), membrane protein (M), and envelope protein (E) [24]. In addition to this, non-structural proteins (Nsp) are also present which are sixteen in total. The spike proteins are the prominent receptor binding sites, where the proteins can interact with receptors present in the body [25]. The S1 subunit includes the receptor binding domain (RBD), wherein the angiotensin-converting enzyme II binds to the spike protein. It also consists of the N-terminal domain (NDT). The S2 subunit possesses the fusion proteins (FP) and transmembrane proteins (TM) [26]. Upon entry of the virus into the host cells, it leads to fusion and uncoating of the viral membrane, followed by transcription and translation of mRNA, which is essential for the formation of viral assembly, which then exoduses out of the host via exocytosis to infect other cells. The entry of viral particles into the other cells of the body leads to stimulation of the immune response and generation of antibodies [27].

**Figure 3:**
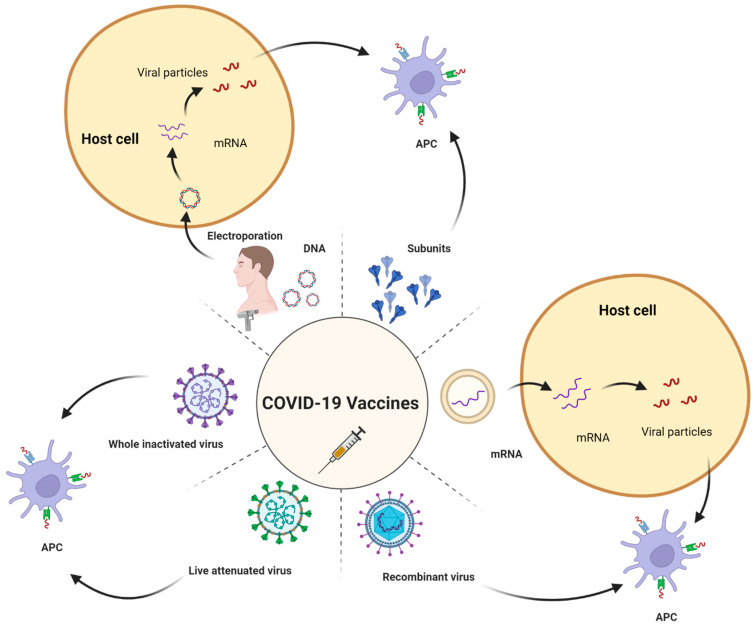
Interaction of vaccines with the host cells and viral particle. (Created with BioRender.com accessed on 31 December 2022).

Globally, immunizations have been widely used and are efficient to decrease mortality rates. Based on sound science and knowledge, the assessment of the safety and efficacy of vaccines and their uptake evaluation can be enhanced [28]. Vaccines have evolved from traditional vaccines such as live attenuated or killed vaccines to subunit vaccines and lastly, homologous or heterologous prime-boost immunization strategies [29]. Different vaccine strategies have been depicted in Figure 3. In antiquity, live attenuated vaccines have been proved to be a saviour against various pathogenic viruses and are considered to be safe against millions of populations, but they are often neglected. Many vaccines including MMR (measles, mumps, rubella) and chicken pox vaccines are live-attenuated vaccines [25,30]. The weakened forms of the virus can be attained by its exposure to adverse conditions or by genetic modifications. This type of vaccine, specifically the oral polio vaccine (OPV) can be administered via non-invasive routes. In contrast, killed vaccines, which are the inactivated versions of the viruses, have limited application in terms of immunological response but the currently used Indian vaccine, COVAXIN^®^, manufactured by Bharat Biotech International Limited, Hyderabad, Telangana, India uses the inactivated version of the virus in its vaccine formulation [31]. The inactivated virus is capable enough to retain its epitome conformity that aids in modulating the humoral immune response [32].

Recent technologies such as mRNA-based vaccines or vector-based vaccines make the most of it for the safety and efficacy of COVID-19 vaccines [33]. The vector-based vaccines are also known as heterologous prime-boost vaccination, which helps in inducing both cellular and humoral immune responses, whereas the live sub-unit vaccines provoke a humoral response only [34,35,36,37,38,39]. According to a study, a 5–10 times increase in T cell-mediated immune response in case of a heterologous vector-based vaccine in comparison to the same formulation, prepared using a homologous approach [40]. Hence, the vector-based vaccines, namely DNA and RNA based are more preferred over sub-unit based, often considered more robust as compared to RNA vaccines, and effective as they encode the spike protein of the virus structure. In the case of the Chikungunya virus, the co-immunization strategy using DNA and virus-like particles generated a better immune response in animal studies [41]. Sometimes, DNA vaccines require specialized delivery systems such as electrophoresis, microneedle arrays, and carrier-based systems such as liposomes to enhance the permeability. Polylactic-co-glycolic acid-based microspheres were formulated for HIV-1 DNA vaccine delivery via the intramuscular route [42]. However, DNA-based vaccines generate a meager immune response and often require another dosing. This can be overcome by the use of mRNA-based vaccines [25,43].

## 3. mRNA-Based Vaccine Development

Formerly, different forms of RNA were used for the vaccine formulation, including in vitro transcribed mRNA, siRNA (small interference RNA), RNA based aptamers, riboswitches, antisense molecules and later-on, mRNA. The awareness of messenger ribonucleic acid, i.e., mRNA originates from its basic, furthermore, economical creation, its transient movement and slow breakdown in the target cells, and its security benefits contrasted with DNA therapeutics as it does not coordinate the human genome, inhibiting insertional mutagenesis, and is promptly accessible for interpretation into protein in the cell cytoplasm [44].

In the early 1990s, researchers worked abundantly to succeed in the development of mRNA-based vaccines. Various experiments were performed in vitro and the results showed a subsequent production of desired proteins; therefore, it was concluded that since mRNA is capable enough to produce proteins, then it can be a probable therapeutic regimen to treat the ailment [45]. According to Martinon et al., the nucleoprotein-based mRNA vaccine for the treatment of influenza was synthesized by in vitro transcription and was incorporated into liposomal vesicles. The results demonstrated the induction of cytotoxic T-cells and antibodies, in vivo [46]. Since then, there has been a progressive development in the use of mRNA as a therapeutic tool due to its significant effectiveness, easy scale-up, and cost-effectiveness, which is a primary requirement for the treatment of highly contagious diseases and cancer therapy. Recent research studies revealed that mRNA-based therapies significantly protected mice from Chikungunya symptoms and maintained desirable levels of antibodies after two doses [47]. RNA-based vaccines target the immune cells of the body by alteration of their functions, targeting the ribosomes for translation, thereby generating an immune response [19].

mRNA-based immunizations comprise single-stranded mRNA, which encodes the antigen desired. They can be conveyed as naked mRNA or encapsulated into suitable carrier systems to facilitate delivery into the cells. Once the mRNA enters the cell, it is translated into protein-based structures by the cell’s natural mechanisms. It is further subjected to post-translational modifications to obtain a protein of interest and generates an immune response as determined by the signal peptides [44,48]. mRNA-based vaccines face a minimum of two hurdles: nuclear degradation when injected into animals and innate immunogenicity similar to that of antigen [49]. To outrange this problem, pseudo-uridine (Ψ) is a well-known RNA modification. Ψ replace uridine with in vitro transcribed (IVT) mRNA, which is highly prevalent, and naturally occurring nucleotide in all the cells with RNAs. Vaccination against COVID-19, produced by both, Moderna and Pfizer-BioNTech consist of novel modified Ψ having efficacy up to 90% against symptoms [50]. Wherein, a vaccine developed by CureVac NV lacked Ψ-modified mRNA and clinical trials confirmed the efficacy of 48% only [51].

**Figure 4:**
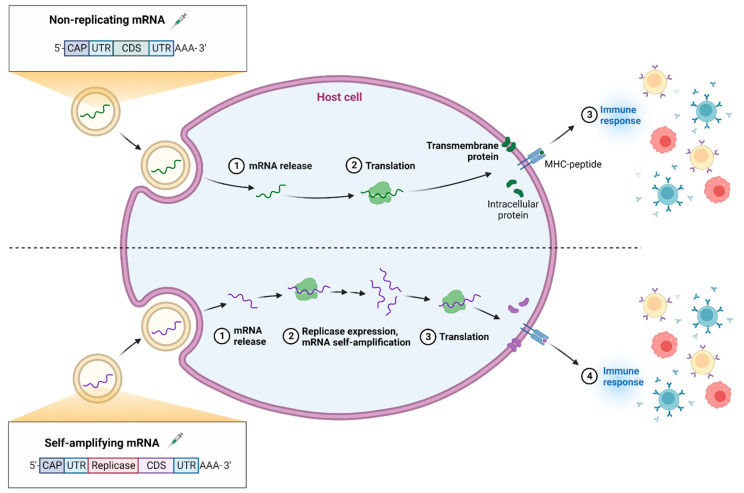
Categories of mRNA. (Created with BioRender.com accessed on 31 December 2022).

**Figure 5:**
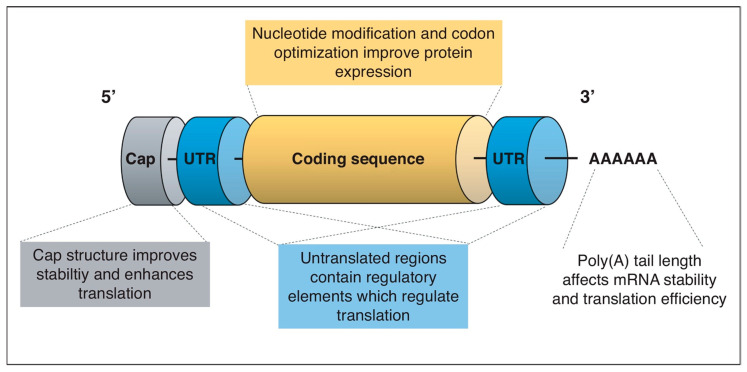
Optimization of mRNA sequence. (Adapted from [56] with rights and permissions).

During the translation process, primarily, two different categories of mRNA vaccines exist, namely, non-amplifying mRNA (NMR) and self-amplifying mRNA (SMR). The working mechanism of non-amplifying mRNA and self-amplifying mRNA is explained in Figure 4. Structurally, as shown in Figure 5, both the structures possess a CAP, 5′ and 3′ untranslated regions (UTR), an opening frame (ORF), and a ponytail in common [52,53].

In addition to this, the SMR possess replicase as a segment which helps in amplification of mRNA intracellularly. Further, the segments are translated by ribosomes in order to produce proteins of interest. The proteins secreted lead to generation of immune response [54]. SMRs are comparatively larger than NMRs and anionic molecules. Recent studies of self-replicating RNA showed better in vitro sub-genome expression of non-structural proteins of Venezuelan equine encephalitis (VEE) replicon when different mutations were observed. A similar clinical trial study was conducted to target the Rabies glycoprotein using VEE-Sindbis virus self-amplifying RNA formulated into nano-emulsions is currently ongoing [55]. The major critical quality attributes (CQAs) which define efficacy of mRNA therapies are summarized in the Table 1 [54].

**Figure 6:**
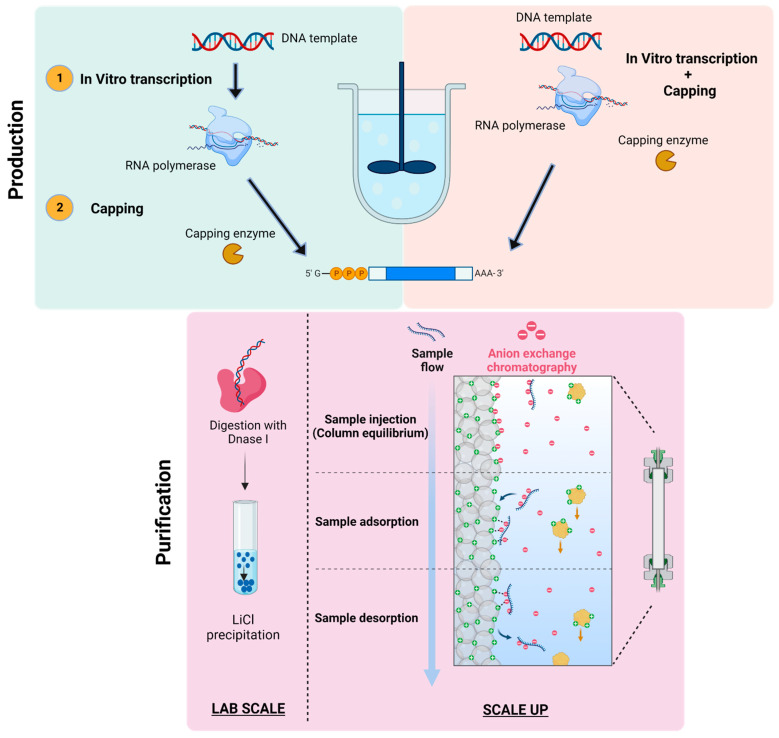
Manufacturing process of mRNA sequence. (Created with BioRender.com accessed on 1 January 2023).

The manufacturing of mRNA is a two-step process, which includes production and purification. No impurities are present in mRNA manufacturing systems as it does not contain any animal or cell-derived raw materials, the production is relatively safer [65]. The production of mRNA sequence may be a single-step enzymatic process or a two-step enzymatic process. A capping analog is used in the case of one-step production process, which is generally used at a laboratory scale. The process is then accomplished by purification, which involves separation using chromatographic techniques as shown in Figure 6. The two-step enzymatic reaction for the production of mRNA encompasses in vitro transcription of the DNA template to form an RNA polymerase sequence [66]. The mRNA template so formed can be capped using vaccinia capping enzyme and methyl donor. The production of mRNA is relatively high and at an industrial scale, it is difficult to achieve reproducibility [67].

## 4. How do mRNA Vaccines Work?

**Figure 7:**
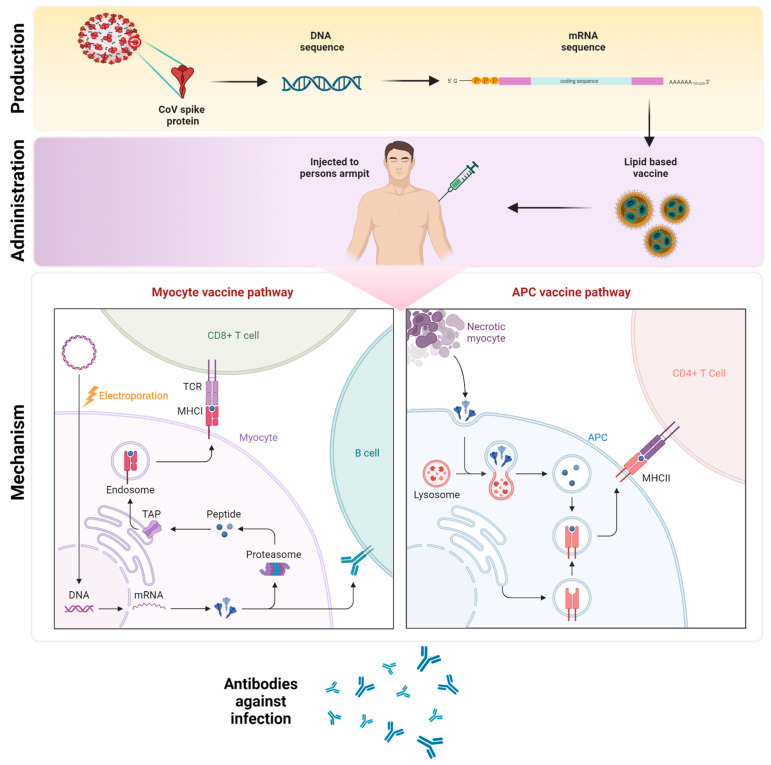
Mechanism through which mRNA Vaccine elicit immunity to the host. (Created with BioRender.com accessed on 1 January 2023).

mRNA is unstable negatively charged molecule which is encapsulated in lipidic nanoparticles. Through endocytosis, these lipidic nanoparticles enter cells. Once enter the cytoplasm these endosomes are immediately directed to lysosomes for degradation. Studies reveal that ionizable lipids play an important role in endosomes’ escape and release of mRNA. The head group of lipids is protonated within the acidic environment of endosomes, and this cationic state is subsequently attached to the anionic head of the phospholipid in the endosomal membrane. The hydrophobic tail of cationic lipid and phospholipid then extends, disrupting the bilayer phospholipid structure and allowing mRNA to escape into the cytoplasmic compartment. Ribosomes transform mRNA into proteins when it is released. This protein can activate an immune response in two ways: (1) The proteasome degrades these proteins into peptides, which are then presented as an antigen on the cell surface by MHC (major histocompatibility complex) class I molecules, which bind to TCR (T cell receptor) to activate CD8+ T cells, which kill infected cells; the protein itself can activate an immune response in two ways. (2) Extracellularly secreted proteins are engulfed by APCs (antigen-presenting cells) and degraded into peptides that are presented on the cell surface by MHC class II and recognised by CD4+ T cells, which secrete cytokines to produce a cellular response and co-activate B cells to produce a humoral immune response, as shown in Figure 7. Furthermore, mRNA vaccines delivered double-stranded and single-stranded RNA that binds to TLR (Toll-like receptor) in the endosome, resulting in the stimulation of numerous IFN-1 (type I interferon) stimulated genes, hence activating antiviral innate immune responses.

## 5. Formulation Aspects of mRNA-Based Vaccines

Once the transcribed mRNA is injected, it will express proteins inside the body. An immunological response is observed due to the expressed protein from the mRNA. This process is predominantly the basis of mRNA vaccine development [68].

### 5.1. DNA Template

Identification of the antigen from the target pathogen is the foremost step. The development of mRNA immunisation needs the addition of the encoded antigen in a DNA template, which will help in the translation of mRNA in vitro. The specific antigen will be produced in vivo by the transcription of the transcribed mRNA, which will only reach the cytoplasm; this will help in eliciting an immune response. The plasmid DNA (pDNA) contains a promoter sequence with strong affinity to a DNA dependent RNA polymerase, such as T7, SP6, or T3, as well as the appropriate sequence for the mRNA construct. The enzyme will pass along the template, extending the RNA transcript until it reaches the template’s end [69].

### 5.2. Capping Enzyme

For efficient translation of mRNA from the DNA template capping at the 5′ end of the mRNA is very much important. Furthermore, 5′ capping in mature mRNA is required for mRNA protection from degradation, gene expression, and ribosome recruitment. A 5′-5′-triphosphate bridge connects the 5′ cap of eukaryotic mRNA to 7-methylguanosine (m7G) (m7GpppN). A novel co-transcriptional technique that employed a clean cap kit to precisely add a natural 5′ cap1 structure to the start site during IVT reactions has made the generation of 5′ capped mRNA by T7 polymerase a commonly used capping method with good translation and minimal reactogenicity. In the COVID-19 BNT162b1 mRNA vaccines, 5′ caps were added using the clean cap method [70]. The cap analog-transcribed mRNA has a poly 1 tail and a 3′(poly A) tail is present at the 3′ end of eukaryotic mRNA. This poly A tail is performing functions of transport, translation, and stability of mRNA. The 3′ poly A tail and 5′ cap will create a stable closed loop structure with the eIF4F complex for the start of the translation process. Because of its 120 nucleotides (nt) in the 3′ poly(A) tail, the BioNTech mRNA vaccine has superior stability and translation efficiency [71].

### 5.3. Nucleotide Triphosphate Substrates (NTPs)

When DNA plasmids are integrated with T7 polymerase and nucleotide triphosphates, mRNA is translated more efficiently and more yields are produced. After administration of the vaccine a concern raised is immunogenicity. On activation of the downstream signaling, the body will recognize and responds to viral RNAs. This recognition of single and double-stranded RNA is by endosomal receptors such as Toll-like receptors (TLR3), TLR7, and TLR8. Double-stranded and 5′-triphosphate modified RNA is recognized by the cytosolic receptor’s retinoic acid-inducible gene-I (RIG-1) and melanoma differentiation-associated protein 5 (MDA-5). Allergic reactions and anaphylactic shock were reported when the body’s immune system is activated in an uncontrolled manner. This overstimulation of the immune system on a molecular level will limit the protein translation, expression of antigens, and vaccine efficacy. This limitation can be overcome by nucleobase modifications. N1-methylpseudouridin, a modified nucleobase, will increase the protein output and decrease the activation of TLR3. Modified nucleotides through RIG-1 not only affect protein-RNA interaction but also decrease the ability of mRNAs to disseminate immunological signals. Translation efficiency of mRNA into protein is improved because of N1-methylpseudouridin. Translation initiation occurring rapidly will coordinately increase the half-life of mRNA [50].

### 5.4. Lipid Nanoparticle Components for mRNA Delivery

**Figure 8:**
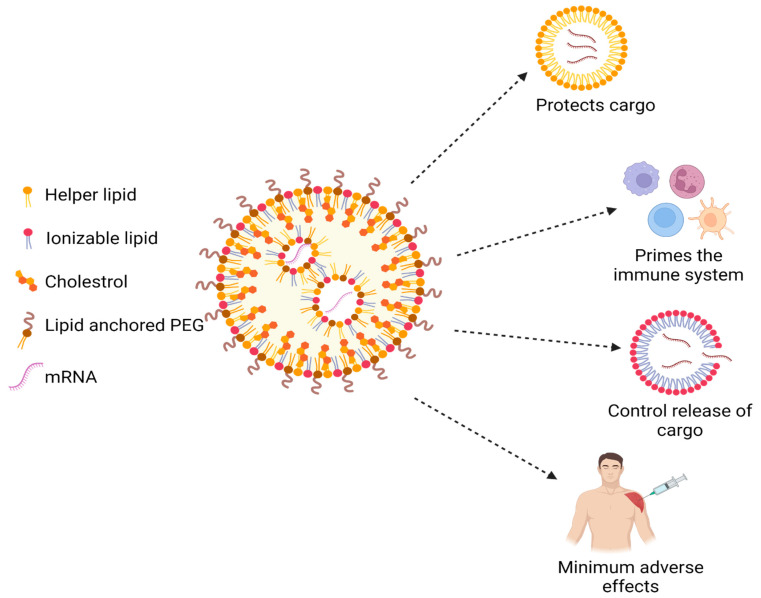
Structure of Lipid Nanoparticles used for efficient mRNA delivery. (Created with BioRender.com accessed on 1 January 2023).

As compared to all other vaccines, mRNA vaccines are developed relatively fast and are more efficacious. However, the greatest challenge in the manufacturing of mRNA vaccines is their poor stability. The vaccine’s stability and capacity to treat the COVID-19 infection are reduced because the vaccine is rapidly broken down by ribonucleases after delivery, producing broken-down components that are eliminated through the kidney. To address this challenge, mRNA can be formulated with lipid nanoparticles (LNPs). The mRNA-LNP vaccine will protect the mRNA from premature degradation and will facilitate cytoplasmic delivery to antigen-presenting cells [72]. Lipids are perfect agents to deliver mRNA at the cytosolic site, as their fusion is compatible with lipid cell membranes; leading to the targeted and effective release of the mRNA. LNPs are nanosized particles with a diameter of less than one micron that contain two or more lipids in varying proportions. LNPs differ from liposomes because of the presence of lipids components in the core as a discontinuous mix. The LNPs that underwent formulation have a multilamellar vesicular structure, a homogeneous core-shell, and a nanostructured core [73]. There is the presence of water inside the core of the LNPs, which exposes mRNA for interaction with an aqueous medium. As shown in Figure 8, mRNA is located inside the LNPs, which protects it from the external environment. The most common composition, which makes up the mRNA-LNP systems are a cationic/ionizable lipid, a phospholipid (“helper lipid”), cholesterol, and a poly-ethylene-glycol (PEG). Physicochemical characteristics inside the LNP delivery system, such as encapsulation effectiveness, outer surface charge, particle size, and shape can be easily modified by adjusting the lipid composition. The components ratios are adjusted depending on the desired target tissue [74].

#### 5.4.1. The Cationic Lipids

With an amine group that imparts a net positive charge, a hydrophobic chain, and a linkage group that enables the attachment of the hydrophilic moiety to the hydrophobic chain, cationic lipids exhibit the amphiphilic property. They have a permanent positive charge at its polar head, which aids in interaction with negatively charged nucleic acids, enhancing the efficiency to entrap. Endosomal escape of the LNP system and cellular uptake is also increased because of the net positive charge. For mRNA administration, cationic lipids, ionizable lipids, and other lipid types have been investigated. As compared to non-ionizable cationic lipids, ionizable lipids, which are positively charged at low pH and at physiological pH remains neutral, are comparatively less toxic due to variations of pH. Inside the cell membrane they are charged positively and in the blood stream they possess uncharged property. BNT162b2 vaccine comprises cationic lipid ALC-0315 whereas mRNA-1273 comprises SM-102; at low pH both of the lipids are protonated which will easily form complex with mRNA. A stream of mRNA in water is combined with a lipid mixture in ethanol using a microfluidic device. In order to trap the negatively charged mRNA the components of these two streams combine quickly to generate nanoparticles [75]. For the BNT162b2 vaccine the molar ratios of the cationic lipid: PEG-lipid: cholesterol: DSPC are (46.3:1.6:42.7:9.4) and for the mRNA-1273 it is (50:1.5:38.5:10). The nanoparticles are having a diameter of 80–100 nm and approximately 100 mRNA molecules are there for one lipid nanoparticle [73].

#### 5.4.2. The Polyethylene Glycol (PEG) Lipids

For stability of colloids and prevention of protein binding to the nanoparticle polyethylene glycol lipids are used. They impart longer systemic circulation by minimizing the clearance of the nanoparticles. LNPs may cause physical aggregation in the solution, this can increase the particle size of the LNPs and PEG lipids can overcome possibly even a premature release of the encapsulated mRNA, this problem. Moreover, the storage stability of the LNPs will also be enhanced [76].

#### 5.4.3. Cholesterol

For transfection of cells and to stabilize LNPs cholesterol is having a vital role. A lower transition temperature is achieved when the contents of cholesterol is increased in LNPs, this will help in releasing mRNA from the LNP and its translocation across the endosomal membrane [77]. In a study conducted by Zhang et al., modified liposomes were formulated using cholesterol-modified cationic peptide DP7 along with Dioleoyl-3-trimethylammonium propane (DOTAP) as a carrier for mRNA delivery. The liposomes were prepared by film hydration technique and then, evaluated for its transfection efficiency. In vitro transfection studies revealed an increase in transfection efficiency with increase in the concentration of DP7 peptide. In addition to this, in vivo anti-tumour studies showed that the complex had better efficacy against the tumour [78].

#### 5.4.4. Other Excipients

The mRNA vaccines are composed of stabilizers eg. Tromethamine, buffers to maintain pH of 7–8, Salts: Helping to balance the acidity in your body, Sugar: Helps the molecules maintain their shape during freezing acting as a cryoprotectant and maintains the long term stability of lipid nanoparticle-mRNA formulation [73]. A summary of key ingredients used in mRNA-based vaccines is provided in Table 2 and Table 3.

### 5.5. Other Delivery System for mRNA

#### 5.5.1. Polymeric Nanoparticles

Polymeric nanoparticles can be synthesized from synthetic or natural polymers such as PLGA [poly (lactic-co-glycolic acid)], chitosan, PLA (polylactic acid), polycaprolactone, gelatin, and poly-alkyl-cyanoacrylates. These polymeric nanoparticles can encapsulate hydrophobic and hydrophilic compounds and proteins, long shelf life; and can modify the delivery of therapeutic compounds. RNA can be encapsulated in self-assembled cationic polymers with hydrophobic modification [80,81].

#### 5.5.2. Cationic Nano-Emulsions

Cationic nano-emulsions have been proven as an effective delivery vehicle for the delivery of nucleic acids. Ccationic lipids present in the nano-emulsions are important for complexes with nucleic acid through electrostatic interactions and improve the transfection efficiency of nucleic acid and its stability. Studies reveal that the mRNA cationic nano-emulsions delivery system enhances immune response by recruiting immune cells and prompting cellular responses to antibodies and T-primates at comparatively low doses [80,82].

#### 5.5.3. Silica Nanoparticles

Mesoporous silica nanoparticles consist of an amorphous silica matrix with well-arranged porosity in the mesoporous range. These nanoparticles have large surface areas with large pore volumes and its surface can be easily modified by certain positively charged moieties to effectively transport negatively charged RNA. Moreover targeted delivery of RNA can be possible by attaching specific ligands on the surface [81]. As per a study conducted by Adam et al., a modified porous silicon microparticle (mPSM)-based nasal vaccine was formulated for COVID-19. The formulation led to increase in helper T cells (Th1) and immune responses and mPSM caused uptake of antigen by nasal pathway. The viral load was found to be reduced significantly [83].

#### 5.5.4. Carbon and Gold Nanomaterials

Carbon nanotubes, Gold nanoparticles, nanographene oxide, and quantum dots, are synthesized nanostructures that also have the potential to deliver RNA to the targeted site and also protecting it from degradation [81].

## 6. Challenges for Storage of Vaccine

The stability of and storage requirements are the major concern for mRNA vaccines. Stability depends on factors such as excipients, pH, and temperature. The COVID-19 vaccine from BioNTech/Pfizer needs to be stored at −80 °C and has a shelf life of up to 6 months, while the COVID-19 vaccine from Moderna needs to be stored at −20 °C and has the same shelf life. BioNTech/Pfizer vaccine requires packaging with dry ice while transporting. Distribution of both vaccines in poor countries of the world is challenging, as mRNA vaccines need to be stored at ultra-cold temperature. These requirements are expensive and in regions of the world with limited resources, arrangements are difficult. The development of a thermostable mRNA vaccine that is clinically efficacious and can be kept for a longer time without incurring large storage costs is one of the problems. According to EMA storage recommendations, mRNA vaccines made by Moderna and BioNTech are stable when frozen for up to 6 months at −25 °C, up to 30 days at refrigerator temperature (4 °C), and up to 6 h at room temperature [84].

## 7. Safety and Efficacy of mRNA Vaccines

### 7.1. Safety of mRNA-Based Vaccines

Assessment of safety related outcomes were carried out for mRNA vaccines. A phase 1 dose-escalation, open-label trial trials of mRNA-1273 was carried out on participants of age between 18 and70 years or older [85]. Study was carried out in two groups in which participants of 18–55 years of age received the vaccine dose of 250 μg and older subjects were given 25 μg or 100 μg of the dose. Solicited local and systemic adverse events, unsolicited adverse events, serious adverse events, and development of new chronic medical conditions were observed after vaccination. The results showed no development of serious adverse events. The most typical adverse reactions reported were headaches, weariness, myalgia, chills, and soreness at the injection site. Neutralizing antibody levels have been demonstrated to correlate with defence against a variety of viruses in humans and with defence against SARS-CoV-2 in animal challenges. The mRNA-1273 vaccine in older adults produced high levels of binding and neutralizing antibodies, and the time- and dose-dependent trends were similar to responses in younger adults. The responses following the second vaccination were comparable to those seen in patients who had recovered from COVID-19 and had donated convalescent serum, including some who were critically ill. Older patients receiving 100 μg dose showed higher antibody and T-cell responses in comparison to those who received 25 μg and the response was identical to the reaction in participants aged 18 to 55 who received the 100 μg dosage. Patients older than 56 years need to receive a second dose of the vaccine to attain neutralizing antibodies. On receiving a booster dose, antibody titers increase rapidly. The safety key results of Moderna and Pfizer vaccines observed during various clinical trials have been listed in Table 4.

After the second dose of the vaccine, local and systemic reactogenicity events increased in frequency and were primarily of moderate severity. The incidence of erythema was found in three participants and lasted for 5 to 7 days. Solicited systemic adverse events such as fever and fatigue occurred in the older subgroup prominently. All these findings suggest that the adverse events solicited or unsolicited were mild to moderately severe. Thus mRNA-1273 was further tested for phase 2/3 trials to assess safety and efficacy in larger populations [95]. Phase 3 clinical trial was a randomized, stratified, observer-blinded, and placebo-controlled study, involving 30,420 volunteers of age 18 years or older, assigned randomly to receive a vaccine (0.5 mL with 100 μg of dose) or placebo. Local reactions for 7 days following immunization, systemic adverse events, unanticipated adverse reactions after 28 days of dose administration, and any significant adverse events were tracked for safety assessment. Solicited adverse effects were predominant in mRNA-1273 in comparison to the placebo group (84.2%, vs. 19.8%). Some of the most observed events were pain at the injection site after vaccination, erythema, tenderness, and induration. These events were cleared up within 4–5 days post-vaccination. After the second dose, the mRNA-1273 group solicited systemic events that became more severe. The presence of hypersensitivity reactions was there in 1.5% and 1.1% of participants in the vaccine and placebo groups, respectively. Other than these complaints, no safety concerns were raised after vaccine administration [106].

A third dose of vaccine was required due to waning protection following two SARS-CoV-2 mRNA injections and the introduction of variations. After administering a third dose of the mRNA vaccine to people who had previously received the mRNA-1273 primary series in the Phase 1 study, their early safety and immunogenicity were assessed. A cross-sectional study performed in Naples (Italy) assessed the preparedness to take the COVID-19 vaccine booster. In this group, the acceptance of the booster dose was nearly 86%. Furthermore, older persons in better health after the main vaccine series, those living with friends or family members who tested positive for COVID-19, and those who had received disease-related information from official public institutions were willing to obtain the booster dose [107].

The bivalent vaccination contained 25 mcg of each mRNA-1273 and mRNA-1273.351, while the booster vaccine formulations contained 100 mcg of mRNA-1273, 50 mcg of mRNA-1273.351, which encodes the Beta variant spike protein. A third dose of the mRNA vaccine showed adequate reactogenicity and safety. When compared to peak responses after the second dosage, vaccination-induced increased in binding and neutralizing antibody titers to D614G, Beta, and Delta variants were similar or larger. It was noticed that neutralizing and binding antibodies reduced constantly. However, detection of antibodies was there at 10–11 months (before third dose) in participants regardless of age and initial primary series dose (e.g., 25, 50, 100, and 250 μg). Injection site pain, weariness, myalgia, and chills were the most often reported adverse effects following the third dosage of the mRNA vaccine, and the frequency of these events was comparable among the three booster vaccine regimens (monovalent prototype, monovalent variant and bivalent groups). With usage of the monovalent or bivalent Beta variant vaccines, no specific advantage or disadvantage was seen in the cellular responses to the epitopes within the Beta-mutated peptides. All of these findings support the current suggestion that a monovalent prototype mRNA-1273 boost be used to provide extensive immunological cross-protection between variants [95].

As per a survey, Heterologous BNT162b2 (BNT) administration in ChAdOx1 (ChAd)-primed participants (ChAd/BNT) demonstrated non-inferior immunogenicity to homologous BNT administration (both prime and booster were BNT vaccines, BNT/BNT), with tolerable reactogenicity and greater T cell responses. In comparison to homologous ChAdOX1 vaccination (ChAd/ChAd), heterologous ChAd/BNT vaccination elicited stronger immunogenicity (ChAd/BNT vs. ChAd/ChAd, antibody titer ratio: 9.2) [108].

A phase 1 trial for BNT162b2 was conducted on 76 participants; they were randomized to receive 10 μg, 30 μg, and 100 μg vaccine doses, and the other group was given the placebo. 58.3% of subjects receiving 10 μg and 100% of 30 μg and 100 μg experienced solicited local reaction pain at the site of the injection after the first dose. The severity of all local reactions was mild to moderate. Fatigue and headache in BNT162b2 were common systemic events. Additionally, chills, muscle pain, and joint pain were also observed. On increasing doses, systemic events were reported more after the second dose was given. A total of 50.0 percent of participants who received either 10 or 30 g of BNT162b1, 58.3 percent of persons who received 100 g of BNT162b1, and 11.1 percent of placebo recipients reported experiencing adverse events. No serious adverse events were reported [109].

In a clinical trial of The Pfizer/BioNTech vaccine (BNT162b2) which was a multinational, placebo-controlled, observer-blinded, randomized, and pivotal efficacy trial, 43,548 participants aged 16 years or older were randomly allocated. A total of 21,720 volunteers were given 30 μg per dose of BNT162b2 and 21,728 with placebo. To determine the safety profile of BNT162b2 characteristics such as pain at the injection site, which is short-term, mild to moderate in case of severity, fatigue, and headache were evaluated. Two injections of BNT162b2 or placebo were given to all the participants, according to randomization 21 days apart, in the deltoid muscle [98]. Local reactions in those who received the BNT162b2 vaccine were reported more as compared to the placebo group. Mild to moderate pain at the site of injection was the most common complaint registered amongst the recipients of BNT162b2 within one week. Severe pain was reported in less than 1% of the population. The pain was observed less in older participants in comparison to that of younger ones. Redness or swelling was less observed. After administration of the second dose, local reactions were less reported [110].

Younger participants who received the vaccine encountered more systemic events than older recipients did. Fatigue and headache (59% and 52%) were the most commonly reported events, and very less vaccinated subjects (<2%) experienced any severe systemic events. After the second dose of the vaccine, 16% of younger groups and 11% of older age reported fever (temperature ≥ 38 °C), which increased the use of antipyretic or pain killers. However, post-delivery of the first dose cases of fever were reported very less [110]. Lymphadenopathy was noticed as an adverse event in BNT162b2 receivers in comparison to the placebo group. A shoulder injury related to the vaccination, right axillary lymphadenopathy, paroxysmal ventricular arrhythmia, and right leg paresthesia were among the unfavourable events recorded among BNT162b2 recipients [98]. Rare adverse effects observed are Bell’s palsy, acute myocardial infarction, cerebral venous sinus thrombosis, pulmonary embolism, Guillain–Barré syndrome, lymphadenopathy, herpes zoster reactivation, stroke, neurological complications, and thrombosis with thrombocytopenia syndrome, and autoimmunity (e.g., autoimmune peripheral neuropathies and autoimmune hepatitis). Among these certain adverse effects such as anaphylaxis, myocarditis, and appendicitis were common in younger people while Guillain–Barré syndrome and myocardial infarction increased with age. These vaccine-associated adverse effects are less frequent than the additional serious adverse effects that occur after severe COVID-19. A recent study reveals the risk of neurological complications in COVID-19 vaccine receivers. The molecular foundation of these adverse effects is unknown. We hypothesize that, since most of these are also apparent in severe COVID-19, it may be associated with acute inflammation produced by both the vaccine and the virus, as well as in the common SARS-CoV-2 S protein. In the BNT162b2 and mRNA-1273 vaccines encoded antigen (S protein) is stabilized it is therefore probable that, if entering the circulation and systemically distributed throughout the body, it can contribute to these adverse effects in susceptible individuals [111].

### 7.2. Efficacy of mRNA Vaccines

For efficacy assessment of mRNA-1273, the vaccine’s primary endpoints were efficacy to prevent symptomatic COVID-19 infection after 14 days of the second dose of vaccine. As a secondary endpoint, prevention of severe COVID-19 infection with persistent symptoms was used. Results after vaccination show that 11 cases of COVID-19 were there in the vaccinated group and 185 cases in the placebo group, indicating 94.1% efficacy of the mRNA-1273 vaccine for treating symptomatic COVID-19. For secondary efficacy endpoints, evaluation of preventing severe COVID-19 was performed. The results indicated 100% efficacy of the vaccine as 30 participants with viral infection were in the placebo group. A study carried out in Qatar showed vaccine efficacy (VE) of mRNA-1273 95.7% against severe, critical, or fatal COVID-19 infection. After ≥14 days of the first dose, effectiveness was 88.1% against B.1.1.7 (alpha) variant and was 100% post second dose. 61.3% and 96.4% of VE were reported after the first and second doses of immunization against the B.1.351 (beta) strain. Therefore, the vaccine is quite successful against both the strain, whether symptomatic or asymptomatic and against any COVID-19 hospitalization and death, even after a single dose [112].

BNT162b2 vaccine’s efficacy (VE) against confirmed COVID-19 having an onset of at least 7 days after the second dose in individuals who were free from virologic or serologic evidence of SARS-CoV-2 infection was the first primary endpoint. The second primary endpoint measured the effectiveness in participants with and without evidence of prior infection and prevention of severe COVID-19 infection. In a research with a total of 36,523 people, 8 instances of COVID-19 with the beginning at 7 days after the second dosage occurred in the vaccination group, compared to 162 cases in the placebo group. This shows 95% of vaccine efficacy. However, the observed VE against the viral disease was 52% between the first and second doses, and it was 91% in the first 7 days following dose 2, attaining complete efficacy against disease with an onset at least 7 days after dose 2. Only 1 of the 10 severe COVID-19 instances that were seen after the first dosage occurred in the group that received the vaccination. This observation is in line with the excellent overall efficacy against all COVID-19 patients. Two cases of infection were there in vaccine recipients amongst hypertension patients and 44 cases in the placebo group. This case reports 94.6% of vaccine efficacy in hypertension patients. Thus BNT162b2 vaccine meets both primary and secondary efficacy endpoints in vaccinated participants [113]. Apart from SARS-CoV-2 infection, with a single dose of the BNT162b2 vaccine, the predicted vaccination efficacy against symptomatic disease with the delta variant (B.1.617.2) was about 36%. After two doses, VE was about 88 percent [114].

## 8. Regulatory Challenges

Regulatory science is the foundation on which regulatory decisions are made. Its primary objective is to develop new methods, tools, and standards for evaluating the safety, efficacy, quality, and performance of regulated products at all stages of their life cycle [115]. mRNA vaccines face the same sorts of regulatory hurdles as any other type of vaccine, including the need to show proof of safety and efficacy in pre-clinical studies, clinical trials, and post-marketing surveillance, as well as maintaining a high standard for the raw materials used in production and consistency of manufacture. Vaccine testing relies heavily on the reliability and reproducibility of enzymes, nucleotides, and linear DNA templates [116]. Raw components, such as lipids, should be strictly regulated, and their purity should be proven. Current good manufacturing practices (cGMPs) should be followed when creating the linear DNA template. To ensure the drug substance is of the highest quality, it is essential to keep an eye on the percentage of capped and polyadenylated mRNA, the number of short transcripts, and the presence of double-stranded RNA. Polyadenylation and capping the 5′ end of mRNA ensure stability and effective translation [21]. The translation efficiency is closely linked to poly (A) tail [114]. Quantifying the amount of mRNA encapsulated in the particle and determining the size distribution of the particles is critical for the drug product because it allows the manufacturer to ensure manufacturing uniformity. Monitoring is typically performed on a number of characteristics, including those pertaining to stability, identity, and sterility. One of the issues for vaccine developers, regulators, and users is the thermal stability of mRNA vaccines [115]. Conventional vaccinations are subjected to stress testing, including prolonged exposure to elevated temperatures, in order to determine their stability under a variety of storage circumstances. This must also be conducted for mRNA vaccines, where it is necessary to evaluate not only the stability of the mRNA molecule but also the preservation of the structure of the vaccine particle itself. Integrity testing protocols for individual mRNA components in multi-component mRNA vaccines are still in need of development. A nucleic acid vaccine’s identification is determined by its sequence. In general, the sequence of the first DNA plasmid used as a template is sufficient for mRNA vaccinations. This is because DNA is instantly translated into RNA. It is not known that sequencing the mRNA, either directly or by converting it to cDNA and then sequencing that DNA, provides any additional information. Even though the error rates of RNA polymerases are substantially greater than those of DNA polymerases, the stated error rates of 1 error every 104–105 nucleotides would be difficult to detect in RNA due to the random nature of the errors. Based on current research suggesting that mRNA vaccines provide protective immunity in animal models, this error rate does not seem to alter the formation of an immunogenic antigen.

Lipid quantification can be conducted by LC-MS/MS [116]. If the protein is translated too quickly, it may not fold appropriately into a functional antigen, which can have the unintended consequence of lowering vaccine efficacy.

## 9. Discussion

In 2021, mRNA vaccines gained attention for their critical position in the pharmaceutical industry’s reaction to COVID-19. Unlike traditional viral vaccines, which use weakened, non-replicating copies of viruses to display antigens and elicit an immune response, the mRNA strategy uses optimized single-stranded mRNA molecules that can provide biological induction. Once you have the vaccine, the cells will take it in, ‘read’ the mRNA sequence, and produce the spike protein. Since your body lacks proteins that resemble that spike, your immune system interprets it as harmful and launches an assault against it. In addition, if you are infected with the coronavirus later, the immune system remembers the spike protein and even recognizes it. The remarkable pace at which the first two COVID-19 vaccines were developed and made publicly available in the USA backs the notion that mRNA vaccines from Pfizer and Moderna received emergency use permission from the FDA in December 2020—less than a year after Chinese scientists announced the coronavirus’s gene makeup. The major benefit of mRNA-based vaccine technologies is their potential to be quickly modified for various diseases since the processing of the target antigen is ‘outsourced’ to host cells, which means that only the genetic code of the antigen is used to design a vaccine candidate. The time between the Chinese government exchanging their SARS-CoV-2 genetic structure and Moderna transporting its vaccine candidate to the National Institutes of Health (NIH) in the United States for phase one studies was just 44 days. mRNA vaccine vaccines have not historically gained regulatory clearance for use in humanity due to a perceived lack of clinical trials for this method, as well as technical challenges related to product stability and distribution. With such an increase in mRNA vaccines in the market, more precise advice to help in the development and assessment of new mRNA vaccines is possible. Control is another field where mRNA vaccine science is in its infancy. There are currently no official guidelines from the US Food and Drug Administration or the European Medicines Agency for mRNA vaccines, even though the drugs are safe enough for major clinical trials.

## 10. Concluding Remarks and Future Prospects

Before 2020, mRNA was not a well-known scientific term in the vaccine world. More than a year since the catastrophic global COVID-19 pandemic began, mRNA-based clinical programs have seen a substantial increase in support, both from the general public and from big pharma. More than 80 million Americans have been vaccinated against SARS-CoV-2-the virus that causes COVID-19, using the game-changing possibilities of mRNA technology. “The vaccine field has been forever transformed and forever advanced because of COVID-19,” says Dan Barouch, MD, Ph.D., director of the Centre for Virology and Vaccine Research at Harvard Medical School. Mostly in a situation of the COVID-19 vaccine, the mRNA strand is engineered to produce the unique coronavirus’s “spike protein,” which produces an immune response that could guard against infection with the actual virus. The vaccines developed by Moderna and Pfizer/BioNTech have been the only mRNA-based vaccines to obtain emergency approval from key regulators, and real-world evidence from the global COVID-19 launch would be critical in vindicating their long-term effectiveness and safety features against the coronavirus and some other viral agents. Exosome-based mRNA delivery will also be the future of mRNA-based therapeutics [117,118]. Mostly with implications of mRNA-based technology becoming apparent as these first-generation vaccines begin to be deployed globally, there is currently a boom in mRNA clinical research for infectious diseases, and industry researchers anticipate an increase in interest in mRNA platforms for other therapeutic fields, especially oncology, but even uncommon autoimmune conditions and neurodegenes.

## Figures and Tables

**Table 1:** CQAs for mRNA vaccines.

CQA	Key Features	Optimization	References
5′ and 3′ Untranslated regions (UTR)	Both 5′ and 3′ UTRs are evident for recognition by the ribosomes and for initiation of translation process and mRNA stability.	Canonical and non-canonical start codons such as AUG and CUG, respectively, must be avoided, which has its influence on the translation process. In an arbitrarily conducted study by Sample et al., 0.3 million random 5′ UTR based predictive model was identified and used to fabricate a new model for optimal protein expression and efficiency.	[57,58,59]
5′ CAP	For effective vaccination mechanisms, 5′ CAP of the mRNA holds as a backbone along with N7-methylated guanosine sidechain. Capped or uncapped mRNAs are identified by Pattern recognition receptors (PRR), which may lead to destruction of mRNA.	Natural capping of the mRNA evades destruction by the PRRs. Dithiodiphosphates are presented in the phosphate bridge, which declines the CAP sensitivity and enhances translation potential. According to a study conducted by Ryzdik, substitution of oxygen in PO4−3 moieties by CCl2 or CF2 resist decapping without diminishing the translation process.	[58,60,61,62]
Poly (A) tail	Usually consists of 15–250 units of adenosine ribonucleotide. It enacts a critical role in regulation of translation and protein expression. The 3′ stretch of the poly tail when attached to a poly(A)-binding protein (PABP) interacts with eukaryotic translation initiation factors (EIFs) and forms a closed-loop assembly.	Addition of a poly-U sequence synergizes the translation process. Pfizer COVID-19 vaccine utilizes a 110-Poly (A) tail consisting of 30 adenosine residues, 10-nucleotide linkers, and 70 adenosine residues. While, Curevac utilizes 64-Poly(A) tail.	[56,63,64]

**Table 2:** Key ingredients used in mRNA-based vaccines.

Ingredients	Functions	References
DNA template	For encoding antigens which will help in transcription of mRNA in the cell.	[66]
Vaccinia-capping enzyme	5′ capping-To cap the mRNA	[67]
Polymerase	T7, SP6 or T3 will mediate synthesis of targeted mRNA form the DNA template	[66]
Nucleotide triphosphates substrates (NTPs)	To improve translation efficiency of mRNA into protein	[69,70]
Lipid Nanoparticle Components	The lipid nanoparticles will enclose the mRNA, which eases uptake of mRNA and endo-cellular penetration to protect against degradation	[79]
Antigen	Full-length S protein with two proline substitutions (K986P and V987P) Wuhan-Hu-1 (GenBank: MN908947)	[75]
Polymerase co factor	MgCl_2_	[75]
Stabilizers	Tromethamine	[75]
Buffer E.g. Potassium dihydrogen phosphate, Disodium hydrogen phosphate dehydrate, Tris (Tromethamine)	To maintain the pH 7–8	[75]
Other excipients A. Salts - Potassium chloride - Sodium chloride - Sodium acetate B. Sugar - Sucrose	Salts: Helping to balance the acidity in the body. Sugar: To maintain the shape of the molecules during freezing.	[75]

**Table 3:** Formulation ingredients of Pfizer and Moderna mRNA vaccines.

Ingredients		Pfizer mRNA Vaccine	Moderna mRNA Vaccine
Active moiety		SARS-CoV-2 viral spike glycoprotein encoded by nucleoside-modified mRNA	SARS-CoV-2 viral spike glycoprotein encoded by nucleoside-modified mRNA
Inactive	Lipids	(4-hydroxybutyl)azanediyl)bis(hexane-6,1-diyl)bis(2-hexyldecanoate) 2[(PEG)-2000]-N, N-ditetradecylacetamide 1,2-Distearoyl-sn-glycero-3-phosphocholine Cholesterol	SM-102 (proprietary to Moderna)PEG 2000 dimyristoyl glycerol 1,2-Distearoyl-sn-glycero-3-phosphocholine Cholesterol
	Other ingredients (Buffers, sugar, diluent, salts)	Potassium chloride, monobasic potassium phosphate, sodium chloride, dibasic sodium phosphate dihydrate, sucrose, sodium chloride	Tromethamin, Tromethamin hydrocholoride, acetic acid, sodium acetate, sucrose

**Table 4:** Safety and efficacy studies of mRNA vaccines in clinical trials.

Brand Name	Company	Clinical Trials Gov. Identifier	Phase of Clinical Trials	Study Design	Race of the Participants	Safety Key Results	Efficacy and Key Results	References
Spikevax (mRNA-1273)	Moderna	NCT04470427	3	Observer-blinded, placebo-controlled (30,000 participants)	High risk COVID-19 patients in the United States	No safety concerns were identified	94.1% efficacy in preventing COVID-19	[86]
		NCT04649151	2	Randomized, Parallel Assignment (4431 participants)	Healthy Adolescents (12 to <18 Years)	Injection-site pain, headache, and fatigue	Not Available	[87]
		NCT04860297	3	Open-label, single intervention study; no randomization (234 participants)	Adult Solid Organ Transplant Recipients	Not Available	Not Available	[88]
		NCT05158140	3	Randomized, Double-blind, Placebo-controlled Study (850 participants)	Booster dose Vaccinated healthy adults (50 Years) or Older aged adults for Pneumococcal Infection	Not Available	Not Available	[89]
		NCT04811664	3	Randomized Controlled Study, Crossover Assignment (1958 participants)	Adults (18–29 years)	Not Available	Not Available	[90]
		NCT04805125	3	Randomized, Parallel Assignment (610 participants)	Immunocompromised patients	Not Available	Not Available	[91]
		NCT05054218		Observational, Cohort (336 participants)	Cancer patients	Not Available	Not Available	[92]
		NCT04405076	2	Randomized, Observer-Blind, Placebo Controlled, Dose-Confirmation Study (660 participants)	Adults (18 Years) and Older	Injection site, headache, and fatigue	Not Available	[93]
		NCT05366322		Cohort, Retrospective (124,879 participants)	Immunocompromised Adults in the United States	It was observed that 2 doses of mRNA-1273 produces lower overall rate of medically-attended COVID-19 as compared to BNT162b2.	Not Available	[94]
		NCT04785144	1	Open-Label, Randomized Study (135 participants)	Naïve and Previously Vaccinated Adults	Well tolerated, safe and acceptable	Not Available	[95]
		NCT04283461	1	Open-Label, Dose-Ranging Study (120 participants)	Healthy participants and Older Adults	Well tolerated, mild or moderate	Not Available	[85,96]
Comirnaty^®^ (BNT 162b2)	Pfizer–BioNTech	NCT04816669	3	Randomized, observer-blind study (629 participants)	Healthy adults (18–55 years)	Not Available	Not Available	[97]
		NCT04368728	3	Multinational, placebo- controlled, observer-blinded, pivotal efficacy trial (45,713 participants)	83% were White, 9% were Black or African American, 28% were Hispanic or Latinx	Administration of two doses of BNT162b2 provided 95% protection against COVID-19 in persons 16 years of age or older.	95% effective in preventing COVID-19 Vaccine efficacy (90 to 100%) was observed across subgroups defined by age, sex, race, ethnicity, baseline body-mass index, and the presence of coexisting conditions	[98]
		NCT04887948	3	Randomized, double blind trial, Parallel Assignment (570 participants)	Adults 65 years of age and older	Not Available	Not Available	[99]
		NCT05308680		Case-control, Prospective study (336 participants)	Israeli health care workers	Local pain at injection site, fatigue, myalgia, sore throat etc.	Increase in serum troponin levels was documented after 4th dose of vaccine in among 0.62% of healthy health care workers	[100]
		NCT04949490	2	Open-label, Rollover Trial (137 participants)	BNT162-04 Trial Subjects	Not Available	Not Available	[101]
		NCT04754594	2	Placebo-controlled, randomized, observer-blind study (349 participants)	Healthy pregnant women	Not Available	96% and 97% effective in documented infection and symptomatic infection (7–56 days), respectively	[102]
		NCT04713553	3	Randomized, observer-blind study (1574 participants)	Healthy participants 18 through 50 years of age	Not Available	Not Available	[103]
		NCT04588480	1	Randomized, placebo-controlled, and observer-blind study (160 participants)	Healthy Japanese adults	No serious adverse events	n the younger and older age groups, respectively, SARS-CoV-2 50% serum neutralizing geometric mean titers were 571 and 366, and geometric mean fold increments were 55.8 and 36.6 one month after dose 2	[104]
		NCT04649021	2	Randomized, double-blind, placebo-controlled trial (950 participants)	Han Chinese adults	Mild or moderate	After one month of vaccination, the neutralizing titer was 294.4 and 5.0 in BNT162b2 and placebo, respectively	[105]

## Data Availability

Not applicable.
